# Supplementary material for: Validation of a Computerized Adaptive Test Suicide Scale (CAT-SS) among United States Military Veterans
Source: PLoS One. 2022 Jan 21;17(1):e0261920. doi: 10.1371/journal.pone.0261920 (PMC8782529; doi:10.1371/journal.pone.0261920)
Supplement: Attachment — Submitted filename: Response to Reviewers 11 2 21.docx [file pone.0261920.s001.docx]

**November 2, 2021**

**Members of the Editorial Board:**

**We are pleased to resubmit the following manuscript entitled: Validation of a Computerized Adaptive Test Suicide Scale (CAT-SS) among United States Military Veterans.** Requests edits/actions are noted below *(italics*) and followed by responses.

1. *It is important that you include a cover letter with your manuscript.  Please ensure that this letter is addressed specifically to PLoS ONE.*

A cover letter that includes the necessary elements has been submitted.

*2. It appears that your ORCiD iD has not been validated in your Editorial Manager account and we are unable to proceed until that step is complete.*

My ORCHID iD is : 0000-0002-2629-214X

I have attempted on a number of occasions to “fetch” my iD so that it can be entered into the system and received the following warning:

I have also sent an email to the editorial office regarding this matter.

On 10/17/21, 5:09 PM, "Brenner, Lisa" <LISA.2.BRENNER@CUANSCHUTZ.EDU> wrote:

Have resubmitted this manuscript - am getting error messages re: my orchid ID. Stating that it is associated with another account. Assistance would be super.

*3. Please include your full ethics statement in the 'Methods' section of your manuscript file. In your statement, please include the full name of the IRB or ethics committee who approved or waived your study, as well as whether or not you obtained informed written or verbal consent. If consent was waived for your study, please include this information in your statement as well.*

The following has been added to the manuscript.

This study was conducted according to the guidelines laid down in the Declaration of Helsinki and all procedures involving human participants were approved by the Colorado Multiple Institutional Review Board (COMIRB).

and

Veterans were eligible if they were between the ages of 18 and 89 and able to provide written informed consent, which was obtained.

*4. We note your updated Data Availability Statement: "Data cannot be shared publicly because of VA policies regarding data and security."*

We have updated our Data Availability Statement.

The full dataset cannot be made publicly available due to privacy concerns and restrictions imposed by the Colorado Multiple Institutional Review Board. All relevant de-identified data are included in the manuscript. For investigators with appropriate authorizations within the Department of Veterans Affairs, requests for data access can be made to [VHAECHMIRECCAdmin@va.gov](mailto:VHAECHMIRECCAdmin@va.gov).

Many thanks for your assistance.

Lisa

Lisa A. Brenner, Ph.D.

Director, VHA Rocky Mountain MIRECC

Vice Chair of Research, Physical Medicine and Rehabilitation (PM&R)

Professor of PM&R, Psychiatry, & Neurology

University of Colorado, Anschutz Medical Campus

Lisa.2.Brenner@cuanschutz.edu

303-724-4588
